# The Italian Spina Bifida registry: insights from the first 1,000 patient data

**DOI:** 10.1186/s13023-026-04526-9

**Published:** 2026-08-04

**Authors:** Marco Salvatore, Adele Rocchetti, Giorgia Buoncuore, Claudia Rendeli, Margherita Capriati, Maria Taverna, Tiziana Redaelli, Alessandra Leo, Raffaella Bruno, Maria Cristina Dieci, Paola Torreri, Spina Bifida registry group, Marco Salvatore, Adele Rocchetti, Giorgia Buoncuore, Claudia Rendeli, Margherita Capriati, Maria Taverna, Tiziana Redaelli, Alessandra Leo, Raffaella Bruno, Maria Cristina Dieci, Paola Torreri, Giuseppe Masnata, Valeria Manca, Flavio Giordano, Asia Sani, Alberto Manassero, Alice Garzotto, Emilio Casolari, Claudia Gatti, Ilaria Rossi, Adriano Ferrari, Silvia Sassi, Elena Corradini, Anna Marucco, Valeria Capra, Gianluca Piatelli, Marco Pavanello, Ferruccio Romano, Laura Grazia Valentini, Arianna Barbotti, Giuseppe Cretì, Rita Perna, Giovanni Mosiello, Giacomo Esposito, Patrizia Pisano, Alessia Peghin, Barbara Tadini, Alice Garzotto, Carmen D’ Aleo

**Affiliations:** 1 National Center Rare Diseases Head - Undiagnosed Rare Diseases Interdepartmental Unit, Istituto Superiore di Sanità, Viale Regina Elena 299, Rome, 00161 Italy; 2 National Center Rare Diseases, Istituto Superiore di Sanità, Rome, Italy; 3 Policlinico Universitario Fondazione Agostino Gemelli, Rome, Italy; 4 Azienda Ospedaliera Universitaria Meyer – IRCCS, Florence, Italy; 5 ASST Grande Ospedale Metropolitano Niguarda, Milan, Italy; 6 ASBI – Associazione Italiana Spina Bifida ODV, Parma, Italy; 7 Spina Bifida Registry Group, Rome, Italy

**Keywords:** Registry, Dataset, Spina Bifida, Natural history, Epidemiologic data, Malformation, Closed spina bifida, Open spina bifida

## Abstract

**Background:**

Spina bifida (SB) results from failure of fusion of the caudal neural tube leading to complications in several organ systems and considerable disability. The causes of this disorder are heterogeneous and multifactorial. Medical and surgical management is a lifelong necessity for people with SB (pwSB) and should be provided by a multidisciplinary team. Standardized collection of data in ad hoc registries represents an important tool to (i) collect clinical and epidemiologic data, (ii) assess the clinical management including the diagnostic delay, (iii) improve patients’ care and (iv) foster research to identify new therapeutic solutions. The present study aimed to shed light on the analyses from the first 1.000 patients included in the Italian SB registry (period 2020-23) thus boosting the contribution of registries of pathology in the improvement of knowledge and research.

**Methods:**

The Italian SB registry was established in 2020 to (i) describe the natural history of the disease, (ii) determine the clinical effectiveness of health care services, (iii) measure and monitor the quality of care of patients. It is based on a web-customizable-based system for the collection of demographics, diagnosis and genetics, patient status, therapy, quality of life and mortality data. Clinical data are updated annually during follow-up visits. Data quality is ensured by an automatic check performed by the system enabling a constant longitudinal and vertical control of data included in the registry. Descriptive statistics, including absolute and relative frequencies, mean, standard deviation, median, and percentiles, were used to summarize the quantitative variables of interest. the Pearson’s Chi-Square Test was conducted to study the association between the qualitative variables under study.

**Results:**

A total of 1.000 pwSB were enrolled in the first 36 months after registry establishment (period: October 2020 to October 2023) from 15 Referral paediatric and adult Italian Centres; 66,8% and 33,2% of patients were enrolled in a paediatric and an adult Centre respectively (53% female versus 47% males). Forty-point-4% (40,4%) of patients was characterized by open spina bifida (OSB) while 59,6% by a closed spina bifida (CSB). Diagnoses were performed during the prenatal period (26,6%), at birth (41,7%) and within the first year of life (15%), most of these latter cases being characterized by a CSB. Associated primary malformations were reported in 57.4% of patients; among these, Arnold–Chiari II malformation was significantly associated with OSB (*p* < 0.001). Lesions were mainly localized in the lumbosacral region (82.3%). Plastic surgery was performed in 549 patients, predominantly among OSB cases (73.5%).

**Conclusions:**

The analyses of our data form, allowed to highlight important demographic and clinical aspects of SB useful to inform future actions to be taken by national health care services and health professionals. The present study represents the first report describing the Italian Spina Bifida Registry, aiming at demonstrating the feasibility and successful implementation of a national registry, which may serve as a foundation for future studies with direct implications for patient care.

**Supplementary Information:**

The online version contains supplementary material available at https://doi.org/10.1186/s13023-026-04526-9.

## Introduction

Neural tube defects (NTDs) are severe congenital malformations affecting 1 in every 1000 pregnancies. Open NTDs result from failure of primary neurulation as seen in anencephaly, myelomeningocele (open spina bifida, SB) and craniorachischisis. SB results from failure of fusion of the caudal neural tube leading to complications in several organ systems and considerable disability; its causes are heterogeneous including chromosome abnormalities, single gene disorders, and teratogenic exposures. Closed spinal dysraphisms comprise a heterogeneous spectrum of congenital anomalies resulting from disturbances occurring during primary neurulation, secondary neurulation, or post-neurulation development. Lesions such as lipomyelomeningocele, split cord malformations, and dermal sinus are typically associated with defects of primary neurulation or premature disjunction between the cutaneous and neural ectoderm, whereas filar lipoma, caudal regression syndrome, and sacral agenesis are more commonly related to abnormalities of secondary neurulation. This distinction also reflects anatomical localization, as lesions situated at or above the conus medullaris are generally linked to primary neurulation defects, while anomalies involving the *filum terminale* more often originate from disturbances of secondary neurulation [1].

A family history of SB or anencephaly is one of the strongest risk factors for these disorders (3% to 8%), in second and third-degree relatives of affected individuals [2].

SB is one of the most complex congenital defects compatible with life; it is associated with malformations of the soft tissues and intrarachidian structures.

Depending on whether intact skin is present on the lesion, SB is classified into open spina bifida (OSB), with lack of skin coverage on the lesion and neural tissue and meninges exposed to the environment; and closed spina bifida (CSB), with intact skin coverage on the lesion. OSB is usually detected by prenatal ultrasound in the second trimester by identifying indirect signs or directly visualizing the spinal lesion; CSB is difficult to detect by prenatal ultrasound and is usually diagnosed in the second trimester or postnatally [3].

Although most studies report lower mortality than all other types of NTDs, first-year- Mortality Rates is approximately 7% for spina bifida, 46% for encephalocele and 100% for anencephaly [4], it is associated with a severe degree of morbidity [5] which can range from being mild to causing serious disabilities including sensory-motor paralysis with neurogenic bladder and bowel, musculoskeletal deformities, associated disorders, and impairment of higher functions.

Prevention of congenital anomalies is a priority issue, representing one of leading causes of infant mortality worldwide [6–8] and involving approximately 1–3% of all live births [9].

In the case of NTD (anencephaly, spina bifida, encephalocele) primary prevention efforts with (i) mandatory fortification of cereal products with folic acid in several countries and (ii) recommendation of folic acid intake for women (400 micrograms per day) who are planning pregnancy or could become pregnant, have led to a widespread decrease in prevalence [10–12].

In Italy, the Folic Acid Promotion Network for the Primary Prevention of Congenital Defects was established in 2004 with the aim of promoting, coordinating and taking more visible actions in favour of an increased intake of folic acid in the peri-conceptional period. As a result, recommendations for the primary prevention of birth defects were published, recommending that women planning a pregnancy, or not actively ruling out this possibility, regularly take at least *0.4 mg per day* of folic acid to reduce the risk of birth defects [13].

The use of rare disease registries and the possibility to collect and share real world data represents an important tool and a useful indicator to estimate the efficacy of healthcare management in preventing congenital defects, thus enabling multidisciplinary collaboration with the overall aim of improving patients’ care [14]. The Joint Research Centre (JRC) of European Commission developed the European Platform for Rare Diseases, aiming at increasing the interoperability of rare disease registries to maximize research results and has published the first practical tool, the “Common Data Elements (CDE) Set for Rare Disease Registration” [15], containing 16 data elements that must be recorded by each rare disease registry in Europe and are considered essential for further research.

The design of the “RegistRare” platform, developed by the National Centre for Rare Diseases (NCRD) at the Istituto Superiore di Sanità (Italian National Institute of Health, ISS), considered the minimum set of common data elements (CDEs), as well as globally validated standards and ontologies. RegistRare aims at collecting clinical and epidemiological data on specific rare diseases in Italy after a formal agreement among all the stakeholders involved in a specific rare disease (namely, patients’ associations, clinicians and researchers) [16].

Italian SB registry was established in 2020 within the frame of RegistRare platform and was developed in collaboration with the Italian SB Patient Association (ASBI, https://www.spinabifidaitalia.it/), the referral SB centres acting within the Italian hospitals and under the coordination of the NCRD at the ISS.

The aim of the present study is to shed light on the important results of the analyses from the first 1.000 Persons with Spina Bifida (pwSB) included in the Italian SB registry (period 2020-23) thus fostering the fundamental contribution of registries of pathology in the improvement of knowledge and research.

## Materials and methods

### Registry scope

The Italian SB registry was successfully launched in September 2020, after being approved by the National ethics committee for clinical trials of public research bodies (EPR) and other national public institutions (CEN) and a Data Protection Impact Assessment (DPIA) is provided. Local ethics committees of clinical partners can contribute to the registry whenever require.

The registry aims to collect information regarding the disease and treatment of all Italian SB pwSB enrolled at the Italian Referral Centres.

Clinical and epidemiologic data are collected to improve and standardize the management of patients at national level, to reduce morbidity and mortality, to accelerate research and to support the development of treatments for SB assessing long-term patient outcomes. Specific aims of the Registry include (i) studying the SB natural history; (ii) better understanding its full spectrum features across the entire population; (iii) identifying unmet medical needs, rare complications, and understudied areas; (iv) facilitating partnerships with stakeholders; (v) expediting completion of clinical trials; (vi) guiding the development of standards of care; and (vii) allowing participants to centrally store their SB medical data.

Additionally, registry aims at stratifying and characterizing SB population, assessing prenatal status and preventive interventions, defining the timing of plastic intervention, estimating the incidence of associated lesions, and evaluating natural and therapeutic outcomes of the disease.

Participation in Registry activities is on a voluntary basis; to date, a total of 15 Italian paediatric and adult referral Centres, representative of the entire country, have been included. At present, there is no definitive estimate of the number of referral centers for spina bifida in Italy. However, it is estimated that the number of centers (spina bifida referral centers) is approximately 40.

### Registry design and protocol

#### Governance

The development of the registry’s IT platform is partially funded by the patient association (namely, ASBI – Associazione Italiana Spina Bifida ODV) thorough the establishment of a dedicated Scientific Collaborative agreement. All subsequent activities, including patient enrollment, data collection, central data collation, analysis, and the preparation of any scientific papers or reports, are carried out on a voluntary basis by all individuals involved in the project, solely for the purposes of scientific research. In addition, all activities of the registry are coordinated by a nominated Scientific Committee (SC) composed by clinicians, researchers, and patients’ association involved, in various capacities, in SB activities at national level. SC is composed by 3 expert members belonging to the patients’ association, 3 researchers from ISS, and 3 clinicians with expertise in the pathology. Its main responsibilities are to organize and oversee the management of the registry. It also defines how to use the data, develops research questions, selects the contents of a dedicated annual report (finely describing all variables included in the registry itself) and decides on internal and external data analysis requests.

#### Dataset

Italian SB registry includes variables from the “Set of common data elements for Rare Diseases Registration” by the European Commission Joint Research Centre [15]; these variables are also provided by EUROCAT [17].

Disease-specific variables were selected and periodically reviewed by the Scientific Committee based on clinical relevance, feasibility of collection, consistency with registry objectives, and alignment with current clinical practice in spina bifida care.

Data collection is performed during the patient’s enrolment; a dedicated and mandatory informed consent is required. Clinical characterization and treatments information are updated during each follow-up visit.

The complete list of variables included and collected within SB registry is described in *Annex 1*; briefly, the following items are collected: enrolment form including demographic information, diagnosis, cause of termination of pregnancy exclusively referred to prior pregnancies reported in the maternal obstetric history, new-born/mother, signs and symptoms, associated conditions, treatments (whenever available), surgeries, death. In particular, (i) demographic and clinical description is evaluated through data on age at diagnosis, type and anatomical level of lesion (OSB/CSB), associated malformations, prenatal and postnatal diagnostic pathways, and longitudinal clinical updates collected during annual follow-up visits; (ii) clinical effectiveness of healthcare services is assessed through variables related to timing and type of surgical interventions, use of prenatal imaging techniques, referral pathways, and management strategies across centres; iii)quality of care is monitored through geographical distribution of patients and centres, inter-regional migration for care and follow-up.

Data collected reflects routine clinical practice and does not require any additional investigations to be carried out. Data collection, upload and follow-up are performed annually through completion of the SB registry platform (namely RegistRare) by authorized medical staff identified at each contributing hospital or referral center with which a specific scientific collaboration agreement is established for the research activities related to the Spina Bifida Registry.

The information entered is derived from hospital medical records. Training of the users is regularly performed, and any eventual query is answered by a dedicated registry helpdesk. User training is provided on a regular basis, and any queries are addressed by a dedicated registry helpdesk.

#### Privacy and security

Italian SB registry is housed within the secure IT Italian National Pathology Registry Platform infrastructure named “RegistRare”. Data are pseudonymised by the site entering the data. All data are stored within this secure infrastructure and can only be accessed by authorised personnel. Data are access is only possible for authorised users with a two-factor authentication. Patients can access the registry exclusively using a qualified e-signature and only for the completion of the section related to quality-of-life assessment. The informed consent form was established in accordance with the relevant regulations and approved by the local institutional ethics committee. The Registry is compliant with Health Information Privacy Laws, the security requirements of the European Union General Data Protection Regulation (GDPR) and the national rules. Data are used (and shared) in an aggregated form and are generally available, after official approval of SC, to all stakeholders to develop projects, policies or studies. Different access levels are defined depending on the stakeholder group applying to use the Registry data.

### Data quality control

Several validation mechanisms have been implemented to ensure the quality of data entry. Measures of assessment at the system input level, including automated quality assurance checks, closed-ended items, validating data types, and mandatory data elements contributed to a general amelioration of completeness of reporting information.

### Patients’ inclusion criteria

The Italian SB registry includes all prevalent and incident cases from pwSB being in follow-up or in consultation. Co-managed patients (any eventual double or triple cases from two or more centers) are identified and excluded by using a dedicated IT system capable of matching the same identification code used for patient anonymization and flagging them during the quality control process.

The Italian SB Registry does not include any age restrictions among its inclusion criteria. Accordingly, both incident cases (new diagnoses) and prevalent cases (including individuals diagnosed in the past) are collected. The registry includes patients of all ages, encompassing both paediatric and adult populations. This approach allows for a comprehensive representation of individuals with spina bifida across the lifespan.

**Fig. 1:**
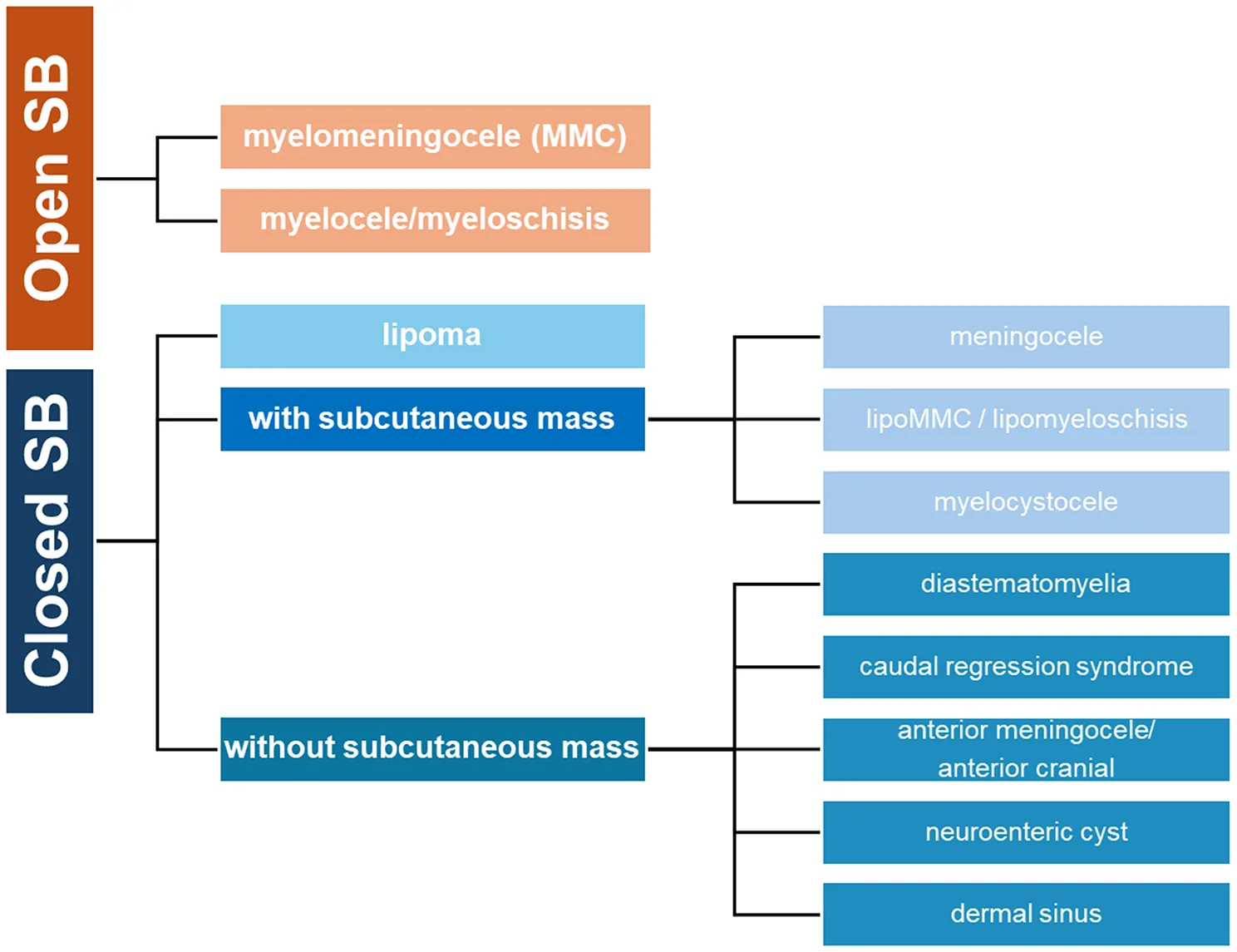
Collection and classification of SB cases (16) *“With subcutaneous mass”* predominantly comprises lipomyelomeningocele (LipoMMC), where the lipoma extends through a posterior spina bifida defect and presents as a visible and palpable external subcutaneous mass, “Lipoma” likely refers to spinal lipomas without an external subcutaneous component, such as filar lipoma or intradural lipoma, which may present without any external mass and are typically diagnosed incidentally or through postnatal MRI

PwSB are collected and classified as briefly exemplified in Fig. 1. So far, both open and closed SB cases are included in the registry. Classification is agreed by all clinicians involved in the activity.

### Registry data access

Internal and external investigators may submit requests for access to aggregated registry data and analysis proposals. All requests are reviewed by the Scientific Committee, which evaluates scientific relevance, methodological appropriateness, consistency with registry objectives, appropriateness of the selected registry variables, data availability, and compliance with ethical and privacy regulations before approval.

### Statistical analysis

Absolute frequencies, percentage frequencies, positional and dispersion indices including mean, standard deviation, median and percentiles were calculated to describe and provide the basic information regarding the quantitative variables of interest (e.g. age at surgery).

The database was then stratified based on the specific analysis subgroups (e.g. diagnosis; period of diagnosis in association with primary malformations in CSB and OSB patients); the Pearson’s Chi-Square Test was conducted to study the association between two qualitative variables (e.g. the association between OSB/CSB specific diagnosis and associated primary malformations).

The resulting p-values ≤ 0.05 indicate the association between variables in analysis. The statistical software used for statistical analysis is SPSS 29.0.1.0.

## Results

### Participating centres

A total of 15 referral Centres were enrolled in the Italian SB registry. Their participation was on a voluntary base. Table 1 summarizes the Centres involved. The participating medical centers cover regions across the country thus representing a collection of data from a nationwide, multicentre large sample of pwSB.

**Table 1:** List of the centres involved in the Italian SB registry activities

Name of the Italian Referral Center	City and Region
AO G. Brotzu	Cagliari (Sardinia, Southern Italy)
AO Meyer	Florence (Tuscany, Central Italy)
AOU Città della Salute e della Scienza - CTO	Turin (Piedmont, Northern Italy)
AOU Parma	Parma (Emilia-Romagna, Northern Italy)
ASST Grande Ospedale Metropolitano Niguarda	Milan (Lombardy, Northern Italy)
AUSL Reggio Emilia	Reggio Emilia (Emilia-Romagna, Northern Italy)
Azienda ULSS 9 Ospedale Legnago	Verona (Veneto, Northern Italy)
IRCCS Istituto Giannina Gaslini	Genoa (Liguria, Northern Italy)
IRCCS Istituto Neurologico C. Besta	Milan (Lombardy, Northern Italy)
IRCCS Ospedale Casa Sollievo della Sofferenza	San Giovanni Rotondo (Apulia, Southern Italy)
IRCCS Ospedale Pediatrico Bambino Gesù	Rome (Lazio, Central Italy)
IRCCS Policlinico San Matteo	Pavia (Lombardy, Northern Italy)
Ospedale Infantile Regina Margherita	Turin (Piedmont, Northern Italy)
Policlinico Universitario Fondazione Agostino Gemelli	Rome (Lazio, Central Italy)
Presidio Ospedaliero S. Elia	Caltanissetta (Sicily, Southern Italy)

Participating centers were identified through the national network of referral centers involved in the diagnosis and management of spina bifida and were invited to participate in the registry on a voluntary basis. The registry currently includes centers with established expertise in the care of individuals with spina bifida across different Italian regions.

### Demography

A total of 1.000 patients were enrolled into the Italian Spina Bifida registry in the first 36 months after registry establishment (period: October 2020 to October 2023).

It is currently not possible to provide a definitive estimation of Italian SB prevalence as the participation in the project is voluntary and has so far involved most, but not all, of the spina bifida diagnostic and care Centres in Italy. Additionally, this estimation is also influenced by significant global heterogeneity resulting from the implementation of health policies based on fortification or recommendation and the type of population under consideration (e.g., live births only versus live births, surviving subjects, and pregnancy terminations). From a comparison with Eurocat database [17] on 2022 data, it is possible to estimate that Italian pwSB is around 6000 people (actual estimated registry coverage 16.7%).

Male vs. female distribution is 46,8% vs. 53,2% with no significant differences (*p* = 0.058). Patient’s age ranged from 0 to 10 years (33,9%), while 32,6% were aged between 10 and 18 years. The remaining 33,4% is included within the second and the seventh decade of life, spanning from 18 years up to 71 years.

Most patients (87.8%) were of Caucasian origin. The remaining participants were of African (6.0%), Hispanic (2.2%), Asian origin (2.2%, including 0.7% of Chinese origin), while 1.1% were classified as having other ethnic backgrounds.

**Fig. 2:**
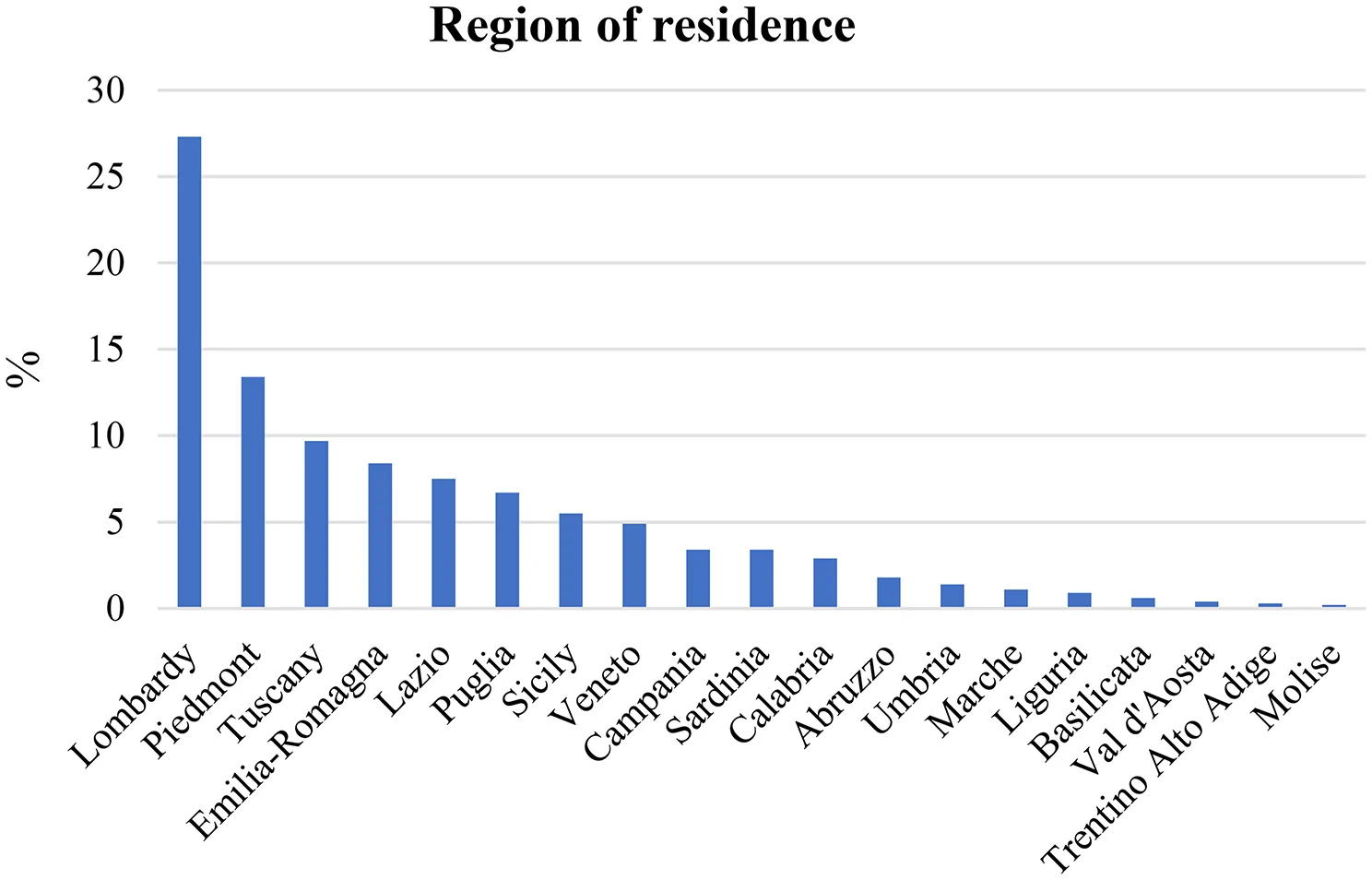
Distribution of patient per region of residence

Figure 2 describes distribution of patient per region of residence; as evident, most part of pwSB is from Northern Italy (40,3% Lombardy and Piedmont).

**Fig. 3:**
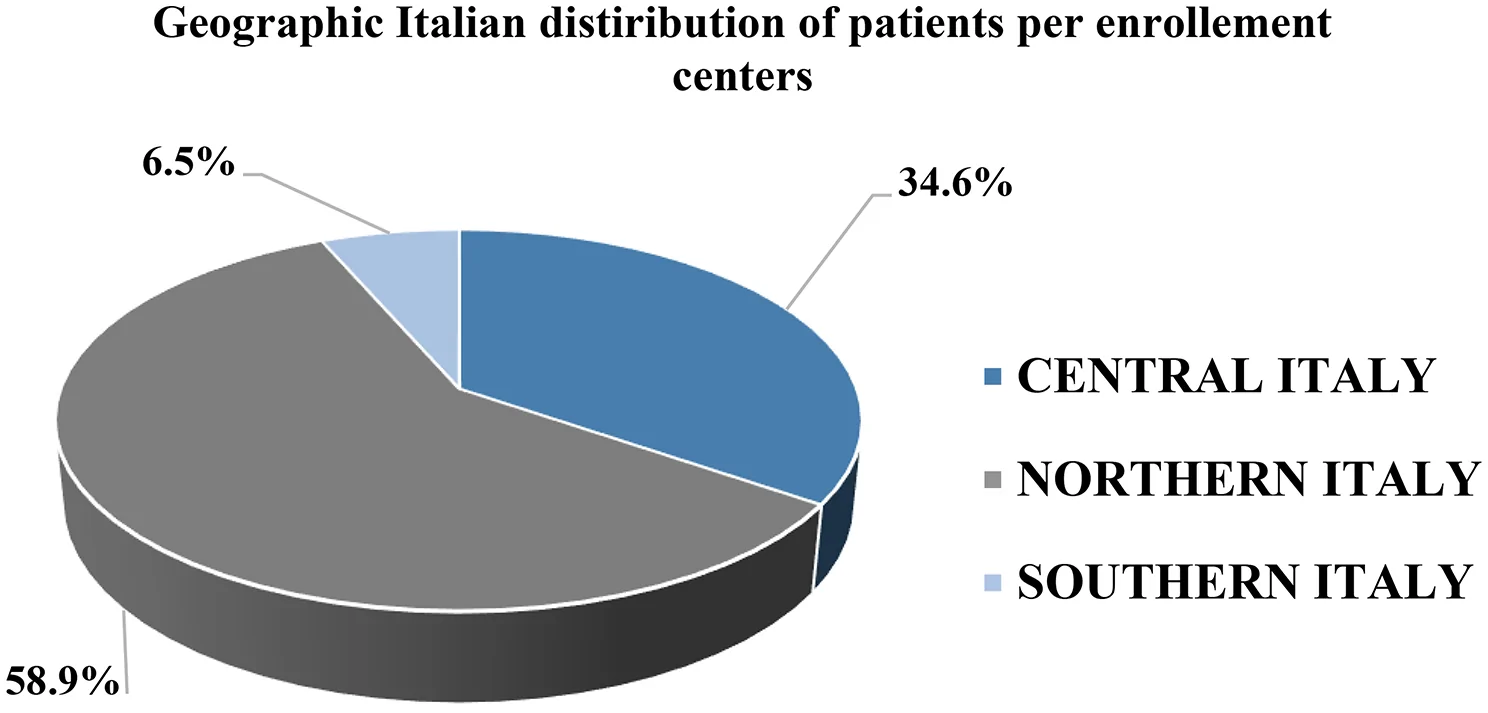
Geographical Italian distribution of pwSB (Northern, Central and Southern). Note: Central Italy includes Lazio and Tuscany regions; Northern Italy includes Liguria, Lombardy, Piedmont, Emilia-Romagna and Veneto regions; Southern Italy includes Puglia, Sardinia and Sicily regions (Italian National Statistics Institute - ISTAT)

Geographic distribution also reflects pwSB distribution per Centre. Figure 3 shows patient’s Italian distribution in Northern, Central and Southern Italy. As shown, most pwSB (58,9%) are enrolled in Centres participating in the Registry’s activities and operating in Northern Italy. This figure clearly reflects the initial limited involvement of Centres in our activity which may not correspond to the actual distribution of patients in Italy.

Furthermore, 28,4% of the patients in the Registry migrate from their region of residence to receive care at a different center. Interestingly, 62,1% of patients were diagnosed in a different Centre from the one where they are taken in care; finally, the 28% receive care in a region different than the one of residence. Data from our registry show that the Italian Regions with the highest rates of healthcare migration are Abruzzi, Basilicata, Calabria, and Campania. Residents from these 4 regions mostly travel to Lazio to receive care: interestingly, the 88.9% of patients from Abruzzo, the 83.3% from Basilicata, the 65.5% from Calabria, and the 50% from Campania are treated in a center located in Lazio region.

### Clinical diagnosis

Our data show that 95,4% of mothers had a natural conception. The median age was 32 years old: respectively 30 years old for OSB and 33 years old for CSB.

Prenatal diagnosis was performed in 26,6% of the patients. Among these, 75,9% were reported to have an OSB, while 24,1% had CSB. Gestational age at suspected diagnosis has been reported at 20 weeks (median age).

**Fig. 4:**
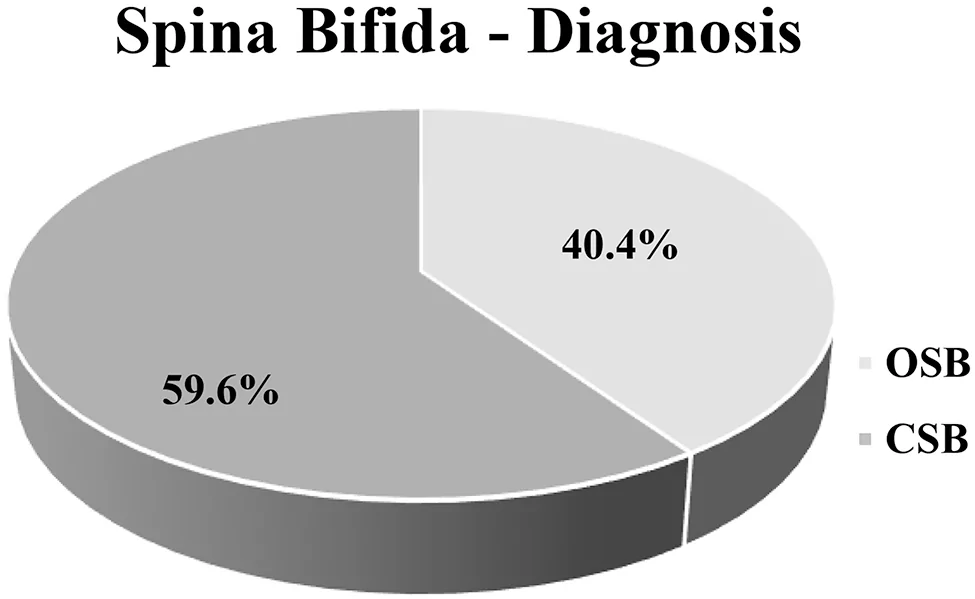
Percentage of pwSB with OSB and CSB

The 40,4% of pwSB are characterized by OSB defect (mainly, myelomeningocele, 99%) while the 59,6% is characterized by CSB (Fig. 4).

Table 2 describes number and percentage of pwSB with CSB: in particular, 36,9% of them was characterized by a subcutaneous mass (mainly, LipoMMC/Lipomieloschisis 82,3%); the 35,7% was characterized by lipoma; the 27,0% was characterized by the absence of subcutaneous mass (mainly, dermal sinus 51,2%).

**Table 2:** Specific characteristics of pwSB with CSB

CSB - Specific Diagnosis	*N*	%
With subcutaneous mass	220	36,9
Lipoma	213	35,7
Without subcutaneous mass	161	27,0
Unknown	2	0,3
**Total**	**596**	**100**,**00**

*“With subcutaneous mass”* predominantly comprises lipomyelomeningocele (LipoMMC), where the lipoma extends through a posterior spina bifida defect and presents as a visible and palpable external subcutaneous mass

*“Lipoma”* likely refers to spinal lipomas without an external subcutaneous component, such as filar lipoma or intradural lipoma, which may present without any external mass and are typically diagnosed incidentally or through postnatal MRI

Lesions are mainly present in the lumbosacral (82,3%) and sacral (13,7%) regions with no significant differences between OSB and CSB. Other lesions are present in dorsal (5,2%), thoracic (1,3%), cervical (0,3%) regions.

As known, NTDs prenatal diagnosis is mainly performed by the simultaneous use of transabdominal ultrasound and specific screening (mainly MRI) aimed at ameliorating diagnosis success rate; indeed, 96,2% of cases are diagnoses by ultrasound and 40,2% by magnetic resonance imaging (MRI) (Table 3).

**Table 3:** Pre-natal test used in the diagnosis of SB

Prenatal test used for OSB and CSB diagnosis	%
Ultrasound	96,2
MRI	40,2
Serum (alpha fetoprotein/other)	2,3
Amniocentesis	7,9
Villocentesis	0,8
Unknown	1,5

Prenatal MRI was performed only in 11,5% of cases to ameliorate NTD definition (Table 4); in particular, 76 cases (out of 115) were characterized by an OSB while 39 were CSB; postnatal MRI was, instead, performed in 82,6% of cases: 304/826 were OSB and 522 were CSB. In 8.9% of cases, this information is not available, as it was not reported by the participating clinical centers. Regarding the remaining 8.5% of patients who did not undergo MRI, the diagnosis was made exclusively on clinical grounds.

**Table 4:** Number and percentage of prenatal and postnatal MRI performed

	Prenatal MRI	Postnatal MRI
	*N*(%)	*N*(%)
Yes	115 (11,5)	826 (82,6)
No	826 (82,6)	85 (8,5)
Unknown	59 (5,9)	89 (8,9)
Total	1.000	

Table 5 shows the percentage of cases for total cases of CSB and OSB per period of diagnosis: most pwSB are diagnosed at birth (41,7%) or pre-birth (prenatal diagnosis, 26,6%). The table also shows the detail for these two groups (at birth and prenatal): CSB diagnosis at birth is 55,9%, while in the case of prenatal diagnosis the majority (75,9%) is OSB. As regards CSB major part of diagnoses is performed after birth.

**Table 5:** Period of diagnosis for total CSB and OSB pwSB

Period of diagnosis	*N* (%)	CSB (% out of *N*)	OSB (% out of *N*)	
Prenatal	266 (26,6%)	24,1	75,9	
At birth	417 (41,7%)	55,9	44,1	
< 1 week	16 (1,6%)	87,5	12,5	
1–4 weeks	37 (3,7%)	97,3	2,7	
1–12 months	97 (9,7%)	95,9	4,1	
Postnatal (age unknown)	22 (2,2%)	81,8	18,2	
> 12 months	128 (12,8%)	97,7	2,3	
unknown	17 (1,7%)	76,5	23,5	
Total	1.000	59,6	40,4	

Table 6 describes results distinguishing case of CSB and OSB pwSB and their respective period of diagnosis. As regards the former, it is interesting to notice that 10,7% of pwSB were diagnosed during prenatal period and 39,1% was diagnosed at birth; 50% of OSB pwSB was diagnosed in prenatal period whereas the 45,5% was diagnosed at birth. Statistical analysis revealed a highly significant association between type of SB (OSB vs. CSB) and period of diagnosis (*p* < 0.001).

**Table 6:** Percentage of pwSB for period of diagnosis of CSB and OSB cases

	CSB	OSB		p-value	
Period of diagnosis	%		%	<0.001	
Prenatal	10,7		50		
At birth	39,1		45,5		
< 1 week	2,3		0,5		
1-4 weeks	6,0		0,2		
1-12 months	15,6		1		
Postnatal (age unknown)	3,0		1		
> 12 months	21,0		0,7		
Unknown	2,2		1		
**Total**	**100**		**100**		

The OSB prenatal diagnoses involve paediatric patients in 67,8% of cases, while the remaining 32,2% refer to adult pwSB with a median age of 24 years and 8 months.

#### Primary malformations associated with SB

An additional primary co-malformation was diagnosed in 57,4% of patients (Table 7) mostly due to a non-genetic cause (89,9%). Table 7 describes the specific associated malformation reported in the registry for both OSB and CSB pwSB. For up to 96% of pwSB (newborn with known mother) a non-association with a NTD was reported and, additionally, for up to 96% of them no previous case of primary malformation has been detected before the diagnosis. Arnold Chiari (type II) is associated with 54,5% of patients. Significative statistical differences were observed between OSB and CSB pwSB as regards several associated primary malformations, such as Arnold Chiari II diagnosis (p-value < 0.001).

**Table 7:** Additional primary specific malformation associated with SB

Associated primary malformations in CSB and OSB pwSB (*N* = 574 out of 1.000)	% (Out of *N*)	CSB (% out of *N*)	OSB (% out of *N*)	*p*-value
Arnold Chiari II	54,5	7,3	92,7	< 0.001
Congenital unilateral or bilateral clubfoot	43,9	24,6	75,4	< 0.001
Strabismus	8,7	22,0	78,0	0.005
Primary malformation of the urinary tract	13,8	68,4	31,6	< 0.001
Aortic coarctation	0,2	0,0	100,0	0.410
Anorectal malformation	9,6	90,9	9,1	< 0.001
Hip dysplasia	20,7	16,0	84,0	< 0.001

### Plastic surgery (spinal cord neurosurgical repair)

A total of 549 plastic surgeries were performed: 73,5% were performed on OSB pwSB, 26,5% on CSB cases. In 8 cases surgery was performed on OSB patients during prenatal period.

Table 8 summarizes age at surgery in CSB patients (including its subtypes).

**Table 8:** Age at surgery in CSB pwSB

	Age at surgery (years, months)						
	Average	Median	Maximum	Minimum	SD	25th percentile	75th percentile
CSB	1,05	0,06	14,00	0,00	2,06	0,01	1,05
With subcutaneous mass	1,02	0,04	13,06	0	2,05	0	0,09
Lipoma	2,05	0,09	14	0	3,06	0,04	3,07
Without subcutaneous mass	1,05	0,11	7	0	1,07	0,05	1,1

Note: Values are reported as years: months (e.g., 1:05 = 1 year and 5 months)

## Discussion

Disease and patient registries are vital tools used to collect, store and manage clinical and genetic data that can be used to monitor diseases, improve clinical best practice, facilitate research, and enable the development of novel therapeutics [19, 20].

Registries for rare diseases are crucial to provide insight into the prevalence and natural history of these disorders, improve diagnostic and management practices, and enable clinical trials of new therapies [21]. Additionally, patient registries are pivotal tools used to monitor disease and facilitate research, and are particularly important for rare disorders, where data collection is challenging due to their low prevalence.

Advances in e-technologies offer new opportunities to collect, integrate and share data and information from different sources and to advance our understanding of diseases thus eventually supporting the development of diagnostic tools as well as new treatments [15]. Registries may use patient-reported data, clinician-reported data or a combination of the two, including several variables and elements ranging from demographic, clinical, genetics, ect. point of view.

RegistrRare is a national registry platform, developed at the National Center for Rare Diseases (Italian Institute of Health) with the specific aim to ameliorate the estimation of epidemiological data (at national level) of specific rare pathologies; its final goal is to override the problem of different registries that may have been individually established at a Centre or at regional level, often dedicated to a single condition and a specific purpose thus ensuring a standardized and quality collection of information at national level.

To date, a total of 14 registries have been developed within this frame [22]. Patients’ associations are strongly involved in the development and management of these registries, designed as a clinician-patient partnership. RegistRare primarily aim is to collect clinical data from patients seen at dedicated referral expert Centres but it also addresses patients’ unmet needs. Patients have a decisional role being part of the Scientific and Technical Committees and, finally, providing an in-kind contribution from sustainability and organisations of registry’s activities.

The choice of data elements is primarily determined by the purpose of the registry itself, including selected variables to improve knowledge on a pathology.

Spina bifida is a rare condition: to date, it is estimated that approximately 6,000 people with SB are living in Italy [17]. Therefore, in order to better define the epidemiology of this condition, the Italian SB Registry was established in 2020. At the moment of the submission of the present paper (April 2025), the results described data from a total of 1.000 pwSB collected between October 2020 and October 2023. Despite the small number of patients currently included in the analyses of the present paper, our aim is to highlight the registry initiative (the first in Italy for this pathology) and to compare our preliminary results with the actual scenario.

Data from our registry are estimated to cover around 16,7% of expected Italian data; the percentage is also expected to increase within the upcoming years due to a more active participation of the Centres involved and operating in Italy. To date, a total of 14 centres from 10 different regions are actively participating to registry’s activities.

Most patients are from northern Italian regions, being the 58,9% of them enrolled in a Centre acting in North of Italy (mainly Lombardy and Piedmont).

About 28% of the patients described in the registry are treated and received care at a center located in a region different from the one they reside in. Data from our registry show that the Italian Regions with the highest rates of healthcare migration are Abruzzi, Basilicata, Calabria, and Campania with around 90% of pwSB residents in Abruzzi region traveling to Lazio Region to receive care. In many cases, SB patients tend to travel to other regions both to access highly specialized referral centers and to seek second opinions or more comprehensive multidisciplinary care. This phenomenon likely reflects not only differences in regional healthcare organizations, but also patients’ perception of quality of life and their preference for centers perceived as offering more specialized and integrated care.

Most patients are characterized by CSB (59,6%) with subcutaneous mass (36,9%), lipoma (35,7%) and without subcutaneous mass (27%). CSB diagnosis is generally performed mostly at birth (39%), while OSB is diagnosed during prenatal period in 50% of cases or at-birth in 45,5% of cases. Diagnosis of OSB is mostly performed during the prenatal period; despite this, our data show that in some cases it was performed also after birth. This is mainly due to the heterogeneity of the population aged between 0 and 71 years, and to the more efficient protocols adopted in the meanwhile.

SB is associated with primary malformation, diagnosed in 57,4% of patients, with most of these defects being due to a non-genetic cause (89,9%).

At present, due to paucity of data, it is difficult to make direct comparisons with registries from other European countries. Nonetheless, our experience represents one of the most innovative initiatives both globally and at the European level. Indeed, as, to our knowledge, only a few similar national registries exist worldwide, including the registry developed in the United States [23, 24]: in particular, US National SB Patient Registry participants ranged in age from birth to 89 years, with 20% aged over 18 at enrollment; most were diagnosed with myelomeningocele (78%), while other forms accounted for less than 6% of cases. Among individuals with myelomeningocele, 77% had a history of shunt placement. The observed difference may be explained by differences in case definitions, inclusion criteria, and the stage of implementation and completeness of the respective registries. In addition, the participating centers may introduce a potential selection bias in data collection, as they are predominantly highly specialized centers for spina bifida care, which may not fully reflect the broader national distribution of cases.

At the European level, the EUROCAT network is active [17]. However, EUROCAT primarily collects demographic and epidemiological data on congenital anomalies, including cases identified at birth, fetal deaths, and terminations of pregnancy for fetal anomaly. Its main objective is surveillance of prevalence trends and support for public health monitoring, rather than the collection of detailed longitudinal clinical data across the lifespan, as in disease-specific registries such as the Italian SB Registry.

### Strengths and limitations

Despite the significant challenges faced during the planning phase of setting up a registry (such as strategies for patient enrolment and retention, limited resources, and voluntary rather than mandatory participation, etc.) [15], the use of a registry remains crucial for collecting clinical and epidemiological data, assessing clinical management, including diagnostic delays, improving patient care, and driving research to identify new therapeutic solutions.

The strength of the SB registry lies in its utilization of data from a nationwide, multicenter cohort of people with SB (pwSB). The participating medical centers span various regions across the country, and the project’s committee includes clinical experts from diverse disciplines, ensuring the reliability and rigor of the data. The data are securely preserved and confidential. In terms of data quality, the variables included align with the European Network of Population-Based Registries for the Epidemiological Surveillance of Congenital Anomalies (EUROCAT) [17], using standardized variable types and values that facilitate cross-regional epidemiological research.

The registry has established a national network of hospitals, effectively gathering demographic and clinical information on pwSB. This will enable large-scale studies on the disease and provide valuable data for governmental bodies and healthcare organizations, informing future prevention and treatment strategies.

As previously stated, this manuscript represents the first report aiming to inform the scientific community about the existence of the Italian Spina Bifida Registry and to promote the involvement and expansion of participating centers in this important initiative. For this reason, we deliberately limited the analysis to a restricted set of variables, specifically those that were most robust and consistently available within the registry at the time of manuscript preparation, and we avoided a direct comparison with other more established and long-standing registries (e.g., the US registry). Within this context, the decision to exclude certain variables from the final analysis, including urinary continence, fecal continence, and ambulation, was made to ensure data completeness and reliability at this early stage of registry development.

A further limitation of this initial report is that formal quantitative assessments of data quality, including data completeness, inter-centre consistency, and source-data validation, are still ongoing and were therefore not included in the present analysis. These aspects will be addressed in future methodological studies as the registry matures and data harmonization across participating centres progresses.

## Conclusion

The present study represents the first report describing the Italian Spina Bifida Registry, aiming at demonstrating the feasibility and successful implementation of a national registry, which may serve as a foundation for future studies with direct implications for patient care.

Italian SB registry collects data from approximately 40% of SB centers present in Italy; epidemiological estimates are restricted to the information available for the first 1,000 patients (approximately the 16.7% of the target population) included in this study. We are confident that our registry offers flexibility through both updatable and longitudinal surveys, allowing for the capture of the natural history of SB, including the onset, severity, and management of its characteristics. We are also confident that, within the next two years, we will include over 6,000 pwSB, thereby expanding monitoring activities and updating the data annually.

We aim at increasing participation of additional Centers by presenting our results during conferences and scientific events, using word-of-mouth dissemination, and by using reports and articles based on the collected data; this will be used to further gain visibility and provide a more concrete and high-quality overview of the work carried out thus far.

Finally, the Italian SB Registry seeks to enhance collaborations with national and international researchers, clinicians, patients, and other stakeholders to advance the understanding of this rare disease, direct efforts in basic and clinical research, and support the development of innovative therapies for this challenging condition.

## Supplementary Information

Below is the link to the electronic supplementary material.

Supplementary Material 1

Supplementary Material 2

## Data Availability

Data sharing not applicable to this article as no datasets were generated or analysed during the current study.

## Abbreviations

SB: Spina bifida
OSB: Open spina bifida
CSB: Closed spina bifida
pwSB: People with Spina Bifida
NTDs: Neural Tube defects
JRC: Joint Research Centre
CDE: Common Data Elements
NCRD: National Centre for Rare Diseases
ISS: Istituto Superiore di Sanità
ASBI: Italian SB Patient Association
CEN: National ethics committee for clinical trials of public research bodies
EPR: and other national public institutions
DPIA: Data Protection Impact Assessment
EUROCAT: European network of population-based registries for the epidemiological surveillance of congenital anomalies
GDPR: General Data Protection Regulation
ISTAT: Italian National Statistics Institute
MRI: Magnetic resonance imaging
