# Supplementary material for: The Italian Spina Bifida registry: insights from the first 1,000 patient data
Source: Orphanet J Rare Dis. 2026 Aug 4;21:391. doi: 10.1186/s13023-026-04526-9 (PMC13732986; doi:10.1186/s13023-026-04526-9)
Supplement: Supplementary file 1 [file 13023_2026_4526_MOESM1_ESM.docx]

**SET OF DATA ELEMENTS INCLUDED IN SPINA BIFIDA REGISTRY**

**LEGEND:** Highlighted variables are in common with both European Platform On Rare Disease Registration (EU RD Platform) [Ref.15] and EUROCAT [Ref.17]

| **ELEMENT NAME** | **ELEMENT DESCRIPTION** | **CODING** |
| --- | --- | --- |
| Surname |  |  |
| Name |  |  |
| Date of birth |  | dd/mm/yyyyy |
| Region of birth |  | Ufficial List |
| Country of birth |  |  |
| Gender | Male - Female - Not known | M/F/NN |
| Ethnicity |  | Caucasian /African /Hispanic /Chinese /Japanese /Asian (not Chinese or Japanese) /other |
| Death before registration |  |  |
| The patient (or parent/guardian) has read and signed the informed consent form |  |  |
| The signed form is kept in accordance with privacy protection regulations under the responsibility of: |  |  |

| **ELEMENT NAME** | **ELEMENT DESCRIPTION** |
| --- | --- |
| **Center of clinical diagnosis** |  |
| **Health care provider** | the center that provides medical care and treatment |
| **Place of residence** | country, region, province |
| **Other registers in which the patient is enrolled** |  |
| **Participation in clinical trials** |  |
| **Biological sample** | Blood, Saliva, urine, cells, cell fractions (DNA, RNA etc.), tissues  Other (free text) |
| **Link to a biobank** | Link to a biobank |
| **Informed consent from patient (parents or legal representative)** | - Enrolment and follow-up data - Patient's permission exists for being contacted for research purposes (clinical trials, biological sample) - Sharing data abroad - Updates on 'secondary' findings |
| **date of consent** | dd/mm/yyyy |
| **date of withdrawal** | dd/mm/yyyy |

**DIAGNOSIS**

| **ELEMENT NAME** | **ELEMENT DESCRIPTION** |  |
| --- | --- | --- |
| **Open spina bifida** | skin does not cover the lesion with external exposure of the meninges and neural placode, occurring anywhere in the column. | - Myelomeningocele (MMC)  - Myelocele / myeloschisis |
| **Closed Spina Bifida** | a defect covered entirely by skin; the spinal cord and the meninges remain in the spinal canal | - With subcutaneous mass  - Without subcutaneous mass  - Lipoma |
|  | with subcutaneous mass | - Meningocele  -LipoMMC / Lipomyeloschisis  - Myelocystocele |
|  | without subcutaneous mass | - Diastematomyelia  - Caudal regression syndrome  - Anterior meningocele = anterior cranial  - Neuroenteric cyst  - Dermal sinus |
| **Anatomical levels of the lesion** | - cervical  - thoracic  - dorsal  - lumbosacral  - sacral  - other (specify) |  |
| **Magnetic resonance imaging (MRI)** | Prenatal (mm/yyyy) |  |
|  | First postnatal (mm/yyyy) |  |
| **Associated primary malformations** | - genetic - non-genetic | - Arnold Chiari II  - Congenital clubfoot mono or bilateral  - Strabismus  - Urinary tract malformation  - Aortic coarctation  - Anorectal malformation  - Hip dysplasia  - Other (free text) |

| **Period of diagnoses** | Prenatal diagnosis  At birth  < 1 week  1-4 weeks  1-12 months  > 12 months  Postnatal diagnosis BUT age unknown  unknown | The first suspected to be malformed |
| --- | --- | --- |
| **Gestational age at which the fetus was first suspected to be malformed** | Number of weeks |  |
| **First positive prenatal test** | - Ultrasound  - Serum (alpha fetoprotein/other)  - Villocentesis  - Amniocentesis  - Karyotype  - Other positive test (text)  - No tests performed  - Not known | first prenatal test whether screening procedure or diagnostic test which indicated a possible congenital anomaly or need for further tests. |
| **Karyotype of infant** | - Performed (text)  - Not performed  -Not known |  |

**DESCRIPTION OF BIRTH/INTERRUPTION, NEWBORN/MOTHER**

**Newborn /fetus**

| **ELEMENT NAME** | **ELEMENT DESCRIPTION** |  |
| --- | --- | --- |
| **Number of babies/fetuses delivered** |  | Singleton  Twins  Triplets  Not known |
| **Malformation in other twins** | Details about twins (heterozygous/monozygous-malformed/non-malformed | Free text |
| Preterm | Yes/no |  |

**Mother**

| **ELEMENT NAME** | **ELEMENT DESCRIPTION** |  |
| --- | --- | --- |
| Age of the mother at delivery | In completed years at the time of delivery. | years |
| Total number of previous pregnancies | The current reported pregnancy is NOT included.  Include all previous abortions whether spontaneous or induced. Multiple pregnancies count as 1 in the total |  |
| Number of miscarriages |  |  |
| Number of induced abortions |  |  |
| Previous malformed siblings |  | Si no non conosciuto |
| Parent’s family with anomalies | Include parents themself | Specify type of anomaly in written text and relation to the infant |
| Parent’s family with NTD | Include parents themself | Specify type of anomaly in written text and relation to the infant |
| Assisted conception |  | - No - Induced ovulation only - Artificial insemination - IVF - GIFT - ICSI - Egg donation - Other - Not known - Assisted conception, type unknown |
| Mother’s occupation at time of conception |  | - employed - unemployed   Not known |
| Assumption of alcohol during pregnancy |  | - Not detected  - Astemium  - Mealtime drinker  -Out-of-meal drinker  - Mealtime and out-of-meal drinker  - consumption > 1lt/day |
| Smoking in pregnancy |  |  |
| Drug taking during pregnancy |  |  |
| Radiation in pregnancy |  |  |
| Folic acid supplementation |  | At least 0.4 milligrams folic acid supplement taken regularly, starting preconceptionally  Folic acid supplement taken irregularly or starting post-conceptionally or at an unknown dose or a dose less than 0.4 milligrams Nessun supplemento di acido folico assunto  No folic acid supplement taken  Folic acid use not recorded for this woman |
| Antiepileptic drug |  |  |
| Drugs | Record any drug taken by the mother | Free text |
| Diabetes |  |  |
| Obesity |  |  |
| Pregnancy weight gain |  | kg |

**SIGNS AND SYMPTOMS**

| **ELEMENT NAME** |  | **ELEMENT DESCRIPTION** |
| --- | --- | --- |
| **Motor level motorio** | - L2 hip flexion  - L3 knee extension  - L4 dorsiflexion foot  - L5 extension I toe foot  - S1 foot plantiflexion  - Paraplegia  - No defcit | Motor level is defined by the most caudal segment in which motor function is preserved with the following key muscles |
| **Neurological bladder** | - Hypertonic  - Hyperactive  - Areflexic  - Dysynergic detrusive-sphincteric  - No defcit | Lower urinary tract impairment secondary to neurogenic damage. |
| **Neurogenic Bowel** | - Obstructive constipation (HP:0012450)  - Fecal incontinence (HP:0002607)  - No defcit | Loss of voluntary control of defecation due to neurogenic-based dysfunction |
| **Congenital deformities** | - spine (HP:0010674)  - hip (HP:0002827)  - knee (HP:0003066)  - foot (HP:0001760)  - No defcit | Alterations of the musculoskeletal system present at birth resulting from a localized and primary error of morphogenesis during the early period of fetal development. e.g., myelodysplastic hip, clubfoot |
| **Acquired deformities** | - spine (HP:0010674)  - hip (HP:0002827)  - knee (HP:0003066)  - foot (HP:0001760)  - No defcit | Musculoskeletal alterations secondary to neurological damage in a morphogenetically intact apparatus e.g., hip and knee flexum, talus foot etc. |
| **Associated conditions** | | |
| **Seizure**  HP:0001250 | age of onset |  |
| **Pressure injuries** (HP:0200042) | - rachis  - sacrum  - lower limbs  - other (free text) | Localized injury to the skin and/or underlying layers, usually at a bony prominence, as a result of pressure, or pressure in combination with shear forces. A tissue injury with necrotic evolution that may involve the epidermis, dermis, and subcutaneous layers, up to and including muscle, cartilage, and bone in severe cases. |
|  | age of onset |  |
| **Pathologic fracture** (HP:0002756)  **lower limb** | - femur  - tibia/perone  - foot  - other (free text) |  |
|  | age of onset |  |

**TREATMENTS**

| **ELEMENT NAME** | **ELEMENT DESCRIPTION** |  |
| --- | --- | --- |
| **Drug** | - anticholinergics - antiepileptics - antibiotics - antispastics - other |  |
| **Bladder catheterization** |  |  |
| **Bladder self-catheterization** |  |  |

**SURGERIES**

| ELEMENT NAME | ELEMENT DESCRIPTION |  |
| --- | --- | --- |
| Prenatal surgery | - Gestational age - name of center - dd/mm/yyyy |  |
| Surgical closure | dd/mm/yyyy |  |
| Ventriculoperitoneal shunting | dd/mm/yyyy |  |
| Endoscopic third ventriculostomy | dd/mm/yyyy |  |
| Surgical decompression of the posterior fossa | dd/mm/yyyy |  |
| tethered cord release surgery | dd/mm/yyyy |  |
| scoliosis surgery | dd/mm/yyyy |  |
| Lower limb deformity correction | - Hip - Feet - Knee - Other (free text) | dd/mm/yyyy |
| Urological surgeries | - Vesicoureteral reflux (VUR) - Bladder augmentation - Ureteropelvic Junction Stenosis - Cryptorchidism (mono/bilateral) - Phimosis - Botulinum toxin - Urinary diversion | dd/mm/yyyy |
| Other | Free text | dd/mm/yyyy |

**DEATH**

| **ELEMENT NAME** | **ELEMENT DESCRIPTION** |
| --- | --- |
| **Date of death** | - dd/mm/yyyy - unknown |
| **Death caused by the disease?** | - Yes/no |
| **Major causes of death** | - other ___________________ - unknown |
| **Comorbidities associated with death** | - Infections - Malignancy - Immune dysregulation - other - unknown |
| **ICD10** | list |
