# Supplementary material for: The Italian Spina Bifida registry: insights from the first 1,000 patient data
Source: Orphanet J Rare Dis. 2026 Aug 4;21:391. doi: 10.1186/s13023-026-04526-9 (PMC13732986; doi:10.1186/s13023-026-04526-9)
Supplement: Supplementary file 2 [file 13023_2026_4526_MOESM2_ESM.docx]

**Spina Bifida Registry Group**

Marco Salvatore¹, Adele Rocchetti², Giorgia Buoncuore², Claudia Rendeli³, Margherita Capriati³, Maria Taverna⁴, Tiziana Redaelli⁵, Alessandra Leo⁵, Raffaella Bruno⁵, Maria Cristina Dieci⁶, Paola Torreri², Giuseppe Masnata⁷, Valeria Manca⁷, Flavio Giordano⁸, Asia Sani⁸, Alberto Manassero⁹, Alice Garzotto⁹, Emilio Casolari¹⁰, Claudia Gatti¹⁰, Ilaria Rossi¹⁰, Adriano Ferrari¹¹, Silvia Sassi¹¹, Elena Corradini¹¹, Anna Marucco¹², Valeria Capra¹³, Gianluca Piatelli¹³, Marco Pavanello¹³, Ferruccio Romano¹³, Laura Grazia Valentini¹⁴, Arianna Barbotti¹⁴, Giuseppe Cretì¹⁵, Rita Perna¹⁵, Giovanni Mosiello¹⁶, Giacomo Esposito¹⁶, Patrizia Pisano¹⁷, Alessia Peghin¹⁷, Barbara Tadini¹⁸, Alice Garzotto¹⁸, Carmen D’Aleo¹⁹.

**Affiliations**

¹ National Center for Rare Diseases, Undiagnosed Rare Diseases Interdepartmental Unit, Istituto Superiore di Sanità, Rome, Italy

² National Center for Rare Diseases, Istituto Superiore di Sanità, Rome, Italy

³ Policlinico Universitario Fondazione Agostino Gemelli, Rome, Italy

⁴ Azienda Ospedaliera Universitaria Meyer – IRCCS, Florence, Italy

⁵ ASST Grande Ospedale Metropolitano Niguarda, Milan, Italy

⁶ ASBI – Associazione Italiana Spina Bifida ODV, Italy

⁷ ARNAS G. Brotzu, Cagliari, Italy

⁸ Azienda Ospedaliera Universitaria Meyer – IRCCS, Florence, Italy

⁹ AOU Città della Salute e della Scienza – CTO, Turin, Italy

¹⁰ AOU Parma, Parma, Italy

¹¹ AUSL Reggio Emilia, Reggio Emilia, Italy

¹² Azienda ULSS 9 Ospedale Legnago, Legnago, Italy

¹³ IRCCS Istituto Giannina Gaslini, Genoa, Italy

¹⁴ Fondazione IRCCS Istituto Neurologico Carlo Besta, Milan, Italy

¹⁵ IRCCS Ospedale Casa Sollievo della Sofferenza, San Giovanni Rotondo, Italy

¹⁶ IRCCS Ospedale Pediatrico Bambino Gesù, Rome, Italy

¹⁷ IRCCS Policlinico San Matteo, Pavia, Italy

¹⁸ Ospedale Infantile Regina Margherita, Turin, Italy

¹⁹ Presidio Ospedaliero S. Elia, Caltanissetta, Italy
